# Big peptide drugs in a small molecule world

**DOI:** 10.3389/fchem.2023.1302169

**Published:** 2023-12-07

**Authors:** Laszlo Otvos, John D. Wade

**Affiliations:** ^1^ Institute of Medical Microbiology, Semmelweis University, Budapest, Hungary; ^2^ OLPE Pharmaceutical Consultants, Audubon, PA, United States; ^3^ Florey Institute of Neuroscience and Mental Health, Parkville, VIC, Australia; ^4^ School of Chemistry, University of Melbourne, Parkville, VIC, Australia

**Keywords:** peptide drugs, small molecules, peptide pharmaceuticals, pharma, peptide drug design and development

## Abstract

A quarter of a century ago, designer peptide drugs finally broke through the glass ceiling. Despite the resistance by big pharma, biotechnology companies managed to develop injectable peptide-based drugs, first against orphan or other small volume diseases, and later for conditions affecting large patient populations such as type 2 diabetes. Even their lack of gastrointestinal absorption could be utilized to enable successful oral dosing against chronic constipation. The preference of peptide therapeutics over small molecule competitors against identical medical conditions can be achieved by careful target selection, intrachain and terminal amino acid modifications, appropriate conjugation to stability enhancers and chemical space expansion, innovative delivery and administration techniques and patient-focused marketing strategies. Unfortunately, however, pharmacoeconomical considerations, including the strength of big pharma to develop competing small molecule drugs, have somewhat limited the success of otherwise smart peptide-based therapeutics. Yet, with increasing improvement in peptide drug modification and formulation, these are continuing to gain significant, and growing, acceptance as desirable alternatives to small molecule compounds.

## Introduction

Almost a decade ago, we summarized the advantages of peptide-based drugs over small molecule counterparts and the necessary target selection, lead optimization and formulation steps required to make synthetic peptide therapeutics both useful and appreciated alternatives from human therapy and pharmaceutical investment perspectives (Otvos and Wade, 2014). Our recommendations included identification of targets to which traditional organic chemistry leads are not available, insufficiently active or induce serious side reactions, taking advantage of the fast peak serum concentration of peptides and favorable medium-term pharmacodynamics parameters. We also described the utilization of innovative sequence modification and formulation strategies to improve their activity profile, *in vivo* half-life and thus mitigate patient compliance disadvantages. In the current report, we highlight the successes (and partial frustrations) of peptide-based drugs from a clinical point of view. A review article from last year lists 36 peptide drugs approved for clinical use since 2000, acting specifically on 26 targets or non-specific biological assemblies (Wang et al., 2022). We have selected 10 peptide drugs from this list that represent various aspects of peptide drug development strategies including both blockbusters and less widely used examples (the word “failure” is not allowed in this review article). The peptide-based drugs (Table 1) are compared with small molecule competitors acting on identical targets or prescribed for the same indications. Note that none of these are considered biologics (in contrast to the insulin family of drugs). According to current Food and Drug Administration (FDA) guidelines, peptides up to 50 residues are regarded non-biological drugs (as opposed to therapeutic proteins, including antibodies and vaccines), mostly driven by the fully synthetic nature of their preparation (Carton and Strohl, 2013). In keeping with the theme of high specificity targets (Kaspar and Reichert, 2013), we do not discuss peptide drugs that act purely via their physical properties (e.g., Lucinactant, formerly known as KL (4) surfactant used for the prevention of neonatal respiratory distress syndrome, Moen et al., 2005), act on complexes of proteins (Carfilzomib (Kyprolis), a proteasome inhibitor against multiple myeloma, Vij et al., 2012), or oligoamides that are recognized as molecular patterns rather than well-defined amino acid sequences (Mifamurtide, a muramyl-dipeptide derivative used for the treatment of osteosarcoma, Myers et al., 2008). Rather, we outline peptide drug development and application concepts instead of an exhaustive review of all approved peptide drugs.

**TABLE 1:** Peptide drugs and their small molecule competitors discussed in detail in this article.

Medical condition	Peptide drug				Small molecule competitor			
	Brand Name	Chemical Name and Primary Sequence	Approval Year	Target	Brand Name	Chemical Name and Structure	Approval Year	Target

HSDD	Vyleesi √	Bremelanotide Ac-Nle-cyclo[Asp-His-*D*Phe-Arg-Trp-Lys]-OH	2019	MCR	Addyi	Flibanserin 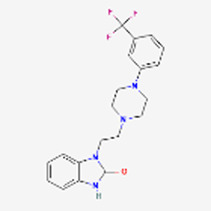	2015	5-HT1A
	
ED	Aviptadil	Zyesami Invicorp H-His-Ser-Asp-Ala-Val-Phe-Thr-Asp-Asn-Tyr-Thr-Arg-Leu-Arg-Lys-Gln-Met-Ala-Val-Lys-Lys-Tyr-Leu-Asn-Ser-Ile-Leu-Asn-NH_2_	2000	VPAC	Caverjet Edex √	Alprostadil PGE1 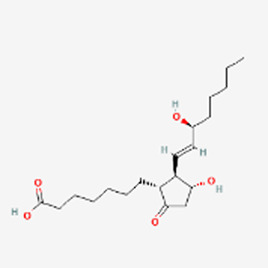	2002	PGR
Type 2 diabetes/obesity	Ozempic Wegovy √	Semaglutide H-His-Aib-Glu-Gly-Thr-Phe-Thr-Ser-Asp-Val-Ser-Ser-Tyr-Leu-Glu-Gly-Gln-Ala-Ala-N6-[N-(17-carboxy-1-oxoheptadecyl-L-α-glutamyl[2-(2-aminoethoxy)ethoxy] acetyl [2-(2-aminoethoxy) ethoxy]acetyl]-Lys-Glu-Phe-Ile-Ala-Trp-Leu-Val-Arg-Gly-Arg-Gly-OH	2017	GLP-1R	Jardiance	Empagliflozin 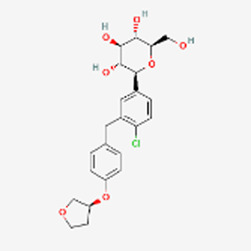	2014	SGLT-2
IBS-C	Linzess √	Linaclotide H–Cys–Cys–Glu–Tyr–Cys–Cys–Asn–Pro–Ala–Cys–Thr–Gly–Cys–Tyr–OH	2012	GC-C	Motegrity	Prucalopride 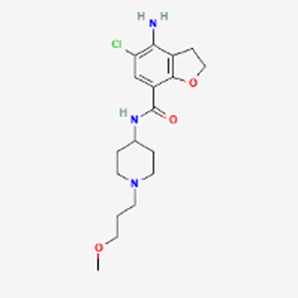	2018	5-HT4
Anemia due to chronic kidney disease	Omontys	Peginesatide 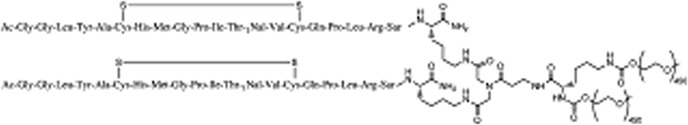	2012; Withdrawn 2013	Erythropoietin receptor				
Acute decompensated heart failure	Natrecor	Nesiritide H-Ser-Pro-Lys-Met-Val-Gln-Gly-Ser-Gly-Cys-Phe-Gly-Arg-Lys-Met-Asp-Arg-Ile-Ser-Ser-Ser-Ser-Gly-Leu-Gly-Cys-Lys-Val-Leu-Arg-Arg-His-OH	2001	NPR-A	Tridil √	Nitroglycerin 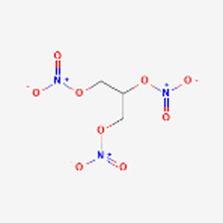	2000	Vascular smooth muscles
Secondary hyperparathyroidism in chronic kidney disease	Parsabiv	Etelcalcetide Ac-*D*Cys (Cys)-*D*Ala-*D*Arg-*D*Arg-*D*Arg-*D*Ala-*D*Arg-NH_2_	2016	CaSR	Sensipar √	Cinacalcet 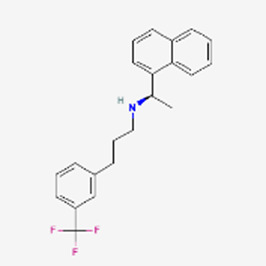	2004	CaSR
AIDS	Fuzeon	Enfivirtide T-20 Ac-Tyr-Thr-Ser-Leu-Ile-His-Ser-Leu-Ile-Glu-Glu-Ser-Gln-Asn-Gln-Gln-Glu-Lys-Asn-Glu-Gln-Glu-Leu-Leu-Glu-Leu-Asp-Lys-Trp-Ala-Ser-Leu-Trp-Asn-Trp-Phe-NH_2_	2003	gp41	Selzentry Celzentry √	Maraviroc 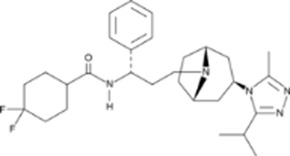	2007	CCR5
Chronic pain	Prialt	Ziconitide H-Cys-Lys-Gly-Lys-Gly-Ala-Lys-Cys-Ser-Arg-Leu-Met-Tyr-Asp-Cys-Cys-Thr-Gly-Ser-Cys-Arg-Ser-Gly-Lys-Cys-NH_2_	2004	N-type calcium channel	None			
HIV-induced lipodystrophy	Egrifta	Tesamorelin Hexenoil-Tyr-Ala-Asp-Ala-Ile-Phe-Thr-Asn-Ser-Tyr-Arg-Lys-Val-Leu-Gly-Gln-Leu-Ser-Ala-Arg-Lys-Leu-Leu-Gln-Asp-Ile-Met-Ser-Arg-Gln-Gln-Gly-Glu-Ser-Asn-Gln-Glu-Arg-Gly-Ala-Arg-Ala-Arg-Leu-NH_2_	2010	GHR	None			

^1^Checkmarks indicate the clinical preference in current medical practice (as seen by these authors).

^2^Please note that with the exception of Etelcalcetide/Cinacalcet, the peptide/small molecule pairs act on different receptors and targets. Accordingly, their binding characteristics cannot be directly compared.

^3^The table shows only the primary peptide sequences. For cysteine bridges in disulfide-containing peptides, please refer to the text. Peginesatide is shown as a full sequence for understanding the spatial contribution of the PEG moiety.

Abbreviations: 5-HT1A, serotonin type 1A receptor; 5-HT4, serotonin type 4 receptor; CCR5, C-C chemokine receptor type 5; CaSR, calcium-sensing receptor; ED, erectile disorder; HSDD, hypoactive sexual desire disorder; IBS-C, irritable bowel syndrome with constipation; GC-C, guanylate cyclase-C; GHR, human growth hormone receptor; GLP-1R, glucagon like peptide 1 receptor; MCR, melanocortin receptors; NPR-A, atrial natriuretic peptide receptor; PGR, prostaglandin receptor; SGLT-2, sodium-glucose cotransporter-2; VPAC, vasoactive intestinal peptide receptor.

### What the doctor will order—with limitations

Peptide drugs are still mostly developed by biotechnology companies at least until late-stage clinical trials (Anand et al., 2023) with big pharma acquiring the products at close-to-market opportunities. The goals of the research process reflect typical biotech thinking: let’s have a receptor antagonist or, better yet, agonist for which no satisfactory small molecule drug is available, select indications where “on demand” injectable therapies with high initial plasma concentration are acceptable such as acutely life-saving or highly desired life-style medications carrying as little side effects as possible. Ease of preparation (low cost of goods) can also be a fringe benefit during the clinical development process.

One of the latest peptide drugs, Bremelanotide (Vyleesi), was approved in 2019 for increasing the sexual appetite of pre-menopausal women with hypoactive sexual desire disorder (HSDD) (Table 1). It is a non-selective agonist of the melanocortin receptors. Bremelanotide is prescribed for women who have not had problems with low sexual desire in the past. The recommended dosage is 1.75 mg injected subcutaneously in the abdomen or thigh at least 45 min before intercourse. The peptide induces improvements in desire, arousal, and orgasm scores (Edinoff et al., 2022). It represents only a very small segment of the drug market with gross product sales to pharmacy distributors for the second quarter of 2022 being $2.3 million, and net product revenue of only $771,000 to Palatin Technologies. Nevertheless, product sales increased by 79% over the prior quarter and by 91% over the comparable quarter in 2021. In 2012, the FDA identified female sexual dysfunction as one of 20 disease areas of high priority and, at the time of approval, considered Bremelanotide a first-in-class medication. The company’s objective is re-licensing the US rights to a committed women’s healthcare company. Bremelanotide is a short peptide without any activity enhancers but carries typical designer peptide drug modifications: a blocked amino terminus, and inclusion of D- and other unnatural amino acid residues. The sequence is Ac-Nle-cyclo [Asp-His-*D*Phe-Arg-Trp-Lys]-OH.

Bremenalotide’s small molecule competitor is Flibanserin (Addyi), a serotonin agonist. New drugs for HSDD were clearly needed, as the FDA had rejected Flibanserin in 2010 and 2013 but succumbed to the pressure and approved it in 2015 although it required several black-box warnings (Sorscher, 2017). While Flibanserin is an oral medication, it mediates a series of drug interactions, particularly with alcohol, central nervous system (CNS) depressants, histamine 2 (H2) blockers and protein pump (PP) inhibitors as well as serotonin uptake inhibitors. It has low efficacy (Dooley et al., 2017) and the manufacturer had legal issues with investors about excessive expectations for activity and business income (Stempel, 2016). The significantly more efficacious peptide interacts only with a very small number of drugs (naltrexone, indomethacin), carries no legal concerns and, assuming that the market share keeps growing at the pace of the first 2 years, can be expected to assume a dominant role in the female HSDD market for quite some time.

A peptide drug for male erectile dysfunction (ED) has traveled a much choppier road. Aviptadil (Zyesami), a human vasoactive intestinal peptide (VIP), was approved in 2000 with phentolamine mesylate for ED (Keijers, 2001). The peptide was originally proposed to treat acute respiratory distress syndrome (ARDS). It is administered as an intracavernosal injection before sexual intercourse. The advent of oral phosphodiesterase-5 inhibitors (Huang and Lie, 2012) and the availability of intracavernosal Alprostadil injections (ICAI) compounded by regulatory issues for Invicorp have limited its use and availability. Currently it is a third line option for men who have failed most non-surgical treatment for ED. The competitor, Alprostadil (prostaglandin E1, Caverject, Edex, Prostin VR), makes blood vessels expand and is similarly administered as intracavernosal injection. When the two drugs are compared, Aviptadil/phentolamine induces a remarkably greater incidence of facial flushing relative to Alprostadil injections (16% versus 3%). Aviptadil is a typical example of unsuccessful peptide drug development. After its little impact as an erectile disorder treatment, during COVID-19 the peptide returned as a potential ARDS treatment as it was initially intended. Given via intravenous injection (a kiss of death), the peptide did not provide any positive primary output measure or anti-inflammatory activity (Youssef et al., 2022) and the FDA declined emergency use authorization in 2021 and 2022 (NRx Pharmaceuticals, 2022). Then a nebulized administration variant was tested in clinically ill patients with COVID-19 in 2022 although no clinical benefits were recorded either. The sequence of the peptide is H-His-Ser-Asp-Ala-Val-Phe-Thr-Asp-Asn-Tyr-Thr-Arg-Leu-Arg-Lys-Gln-Met-Ala-Val-Lys-Lys-Tyr-Leu-Asn-Ser-Ile-Leu-Asn-NH_2_.

### Whoa, ho ho, it’s magic, yesss

The past decade has witnessed the rise of several blockbuster peptide drugs. When Pilot, a one-hit-wonder Scottish rock band, patented the song It’s Magic in 1974 (Whoa ho ho ho, it’s magic), nobody ever thought that this would be the most broadcast tune in 2023 on US television. This remarkable success came from the commercial remake of the song to advertise Ozempic (Semaglutide, Whoa ho, ho, Ozempic), a GLP-1 analog (Mahapatra et al., 2022). GLP-1 agonists stimulate glucose-dependent insulin release from pancreatic islets as their primary effect (Lee and Jun, 2014). Other main effects are to slow gastric emptying and to reduce appetite (Nauck et al., 1997). According to the Centers for Disease Control and Prevention (CDC), about 35 million Americans suffer from type 2 diabetes. This figure is 460 million patients worldwide, about 6.3% of the total population. Just to show how big a business the production of anti-diabetic drugs, it is enough to say that the worldwide incidence of all cancer types is 1.3% (Roser and Ritchie, 2015). Native incretin peptides were originally not suitable for drug development but the enormous need for an anti-diabetic drug forced research to make GLP-1 receptor agonists ultimately druggable (Todd and Bloom, 2007). For Semaglutide, the tide-turning substitutions are a glycine → 2-aminoisobutyric acid (Aib) replacement at position 2 and the attachment of an octadecanoic diacid to the side chain of Lys-26. The peptide chain modifications render the native peptide increasingly resistant to the proteolytic enzyme DPP-4, and the presence of the fatty acid moiety results in a high binding affinity to serum albumin increasing the half-life to approximately 7 days in humans (van Witteloostuijn, et al., 2016). Semaglutide, built on a human GLP-1 backbone, was initially approved in 2017, is administered at 0.25 mg subcutaneously weekly. Other GLP-1 receptor peptide agonists include Dulaglutide (Trulicity, injected weekly); Liraglutide (Victoza, Saxenda, daily) both also built on the human GLP-1 backbone, and Exenatide (Byetta, twice daily), Exenatide extended release (Bydureon bcise, weekly), Lixisenatide (Adlyxin, daily), the last ones being built on the exendin-4 backbone. Novo Nordisk, the manufacturer of Semaglutide, has rapidly grown so large that the company is reshaping the Danish economy. Ozempic and Wegovy (the latter contains higher doses up to 2.4 mg of Semaglutide and is designed for weight loss) have been proclaimed as revolutionary in the field of obesity. Ozempic and Wegovy sales are $12 billion and $4 billion a year respectively. Denmark’s recent economic growth can be attributed solely to Novo Nordisk’s booming success. In the past few weeks, Novo Nordisk’s market value has exceeded the size of the entire Danish economy (Nelson, 2023). Part of Wegovy’s success is the auto-injection device that is a piece of art. The patient does not even see the needle or needs to engage a frightening button. Just push the drug autoinjector tube against the abdomen and wait until the color gauge indicates complete loading of Semaglutide. The Semaglutide sequence is: H-His-Aib-Glu-Gly-Thr-Phe-Thr-Ser-Asp-Val-Ser-Ser-Tyr-Leu-Glu-Gly-Gln-Ala-Ala-N6-{N-(17-carboxy-1-oxoheptadecyl-L-α-glutamyl [2-(2-aminoethoxy)ethoxy] acetyl [2-(2-aminoethoxy) ethoxy]acetyl}-Lys-Glu-Phe-Ile-Ala-Trp-Leu-Val-Arg-Gly-Arg-Gly-OH.

The non-peptide competitor of Ozempic is Jardiance (Empagliflozin), a sodium glucose co-transporter 2 (SGLT-2) inhibitor. SGLT-2 inhibitors are used to lower high blood glucose levels in people with type 2 diabetes. They may also be called gliflozins. Jardiance dose is a 10 mg tablet daily. Its sales are $6 billion a year, nothing to sneeze at, yet significantly below the sales of Semaglutide. This is probably due to better patient experience with the peptide than with its small molecule partner. According to Drugs.com, the overall user rating of Ozempic is 6.0, with 46% positive and 32% negative experience. The values for Jardience are 5.2 overall, 39% positive and 43% negative. Other approved small molecule SGLT-2 inhibitors include Brenzavvy (Bexagliflozin), Invokana (Canagliflozin) and Farxiga (Dapagliflozin).

Ozempic/Wegovy achieved blockbuster status through extended activity changes and a patient compliant administration mode, a quasi-oral injectable strategy. Yet, the final goal of all peptide drugs is truly oral administration and Semaglutide is not an exception. Clinical trials with 25–50 mg Semaglutide pills are in progress (Aroda et al., 2023; spoiler: good efficacy but 80% of patients reported adverse effects). In contrast, another huge peptide drug owes its success to the lack of adsorption after oral administration. Linaclotide (Linzess) is a guanylate cyclase 2C (GC-C) agonist (McCormack, 2014). It is a first-in-class agent approved in 2012 for the treatment of adult patients with moderate to severe irritable bowel syndrome with constipation (IBS-C). Linaclotide does not get absorbed in the gastrointestinal tract; rather it binds the receptor on the intestine surface. Upon binding, the peptide stimulates fluid secretion, increases colonic transit, and reduces abdominal pain (Camilleri, 2015). The daily oral dose is 0.15–0.3 mg. Linaclotide is an unmodified *Echerichia coli* enterotoxin ST sequence. H–Cys–Cys–Glu–Tyr–Cys–Cys–Asn–Pro–Ala–Cys–Thr–Gly–Cys–Tyr–OH, Cys bridges between residues 1–6, 2–10, 5–13. The low dose needed reflects Nature’s evolutionary pressure to optimize the native peptide for its biological function i.e., travelers’ diarrhea. Linzess’ annual sales reached $1 billion in 2021 which is an impressive figure especially if we consider that the treatment success is not a television-friendly result (as opposed to the addictive Ozempic tune) for commercials. Patients in Linzess television commercials do express verbally what most of us do after a productive morning event, yesss. Linaclotide’s peptide sequence is somewhat similar to that of Plecanatide. Plecanatide is sold under the trade name Trulance. Plecanatide is uroguanilin, a hexadecapeptide, expressed by a gain-of-function mutation of GC-C and similarly prescribed for chronic idiopathic constipation (CIC) and IBS-C in adults (Kamuda and Mazzola, 2018).

The major non-peptide competitor of Linzess is Motegrity (Prucalopride), a selective serotonin type 4 (5-HT4) receptor agonist against CIC. Motegrity at a daily dose of 2 mg sells at a dramatically lower volume of $75 million annually. Currently no generic variant is available for either medication. Linzess’ 30 day costs are $400; with health insurance, $10. Once again, the peptide drug enjoys its business success due to patient preference. The overall ratings at Drugs.com are 6.6 for Linzess and 5.4 for Motegrity, with 14% more patients reporting positive experience and 14% less reporting negative experience with the peptide compared to the non-peptide competitor.

### Lessons learned from other peptide drugs

Peptide drug developers must be very cautious with the selection of covalent additives or formulation enhancers. Peginesatide (Omontys) is a pegylated peptide against anemia due to chronic kidney disease. It is a dimer of the erythropoietin-mimetic peptide (EMP-1) that activates the erythropoietin receptor but bears no resemblance to erythropoietin (Wrighton et al., 1996). As EMP-1 had no potency, a dimeric peptide was made and conjugated to polyethylene glycol (PEG) to enhance the half-life to 50 h after intravenous administration (McDougall et al., 2009). The final composition is a heterodetic cyclic peptide composed of two identical 21-amino acid cyclic peptide units (including 3 non-natural residues) covalently bonded via a linker derived from iminodiacetic acid and β-alanine to a lysine-branched 40 kDa poly (ethylene glycol) chain. Omontys was approved in 2012. However, in Phase IV clinical trials, serious adverse drug reactions (sADRs) were reported including not only cardiovascular events but actual mortality (Hermanson et al., 2016). The approval was withdrawn in 2013. When the causal factors of the sADRs were investigated, experts pointed to the conjugated polyethylene glycol moiety. Soon after the withdrawal of Omontys, a large clinical trial was terminated early due to anaphylaxis following intravenous administration of REG1, a novel anticoagulation system containing a synthetic pegylated anticoagulant (Lincoff et al., 2015). In general, since the time of the Peginestide saga, it has been recognized that pegylation of polyamide therapeutics candidates is not without potentially game stopping disadvantages (Zhang et al., 2014).

Pharmacoeconomical considerations can sink otherwise promising peptide dugs. Nesiritide (Natrecor) is a human B-type natriuretic peptide approved in 2001 against acute decompensated heart failure. H-Ser-Pro-Lys-Met-Val-Gln-Gly-Ser-Gly-Cys-Phe-Gly-Arg-Lys-Met-Asp-Arg-Ile-Ser-Ser-Ser-Ser-Gly-Leu-Gly-Cys-Lys-Val-Leu-Arg-Arg-His-OH (disulfide bridge between the two cysteines). The target of the peptide is the natriuretic peptide receptor. It is a relatively long and complex peptide and competes with the non-peptide drug Nitroglycerin, arguably the simplest and probably least expensive small molecule medication of all. In acute (as opposed to chronic) heart failure, both drugs are administered intravenously. When added to standard care in hospitals, the peptide improves hemodynamic functions better than Nitroglycerin. Pulmonary capillary wedge pressure decrease (the standard readout of heart failure therapy efficacy) in 3–24 h is 25%–60% larger with Nesiritide compared to Nitroglycerin (Young, 2002). However, Nesiritide treatment (drug price plus continuous infusion, 2 days more treatment time) costs 40 times more than that of Nitroglycerin. Hospitals and insurance agencies agree that improvements in mortality and morbidity upon peptide administration do not warrant the considerably higher medical costs. The recommendations suggest that, in spite of the better clinical efficacy, the peptide should not be considered as first line therapy (Noviaski et al., 2003).

While it is a common trend to replace marketed peptide therapeutics with competing small molecule drugs, it does not seem to be a good idea to go the other way around. Cinacalcet (Sensipar) is an orally administered non-peptide allosteric modulator of the calcium sensing receptor (Balman Balfour, and Scott, 2005). CaSR is predominantly expressed in the parathyroids and kidneys (Thakker, 2012). Cinacalcet was approved in 2004 and is marketed by Amgen. Sensipar (cinacalcet) sales are now under $100 million annually. The sales recently decreased 70% year-over-year, primarily driven by volume decline in response to generic competition. Amgen purchased a company selling a peptidic direct CaSR agonist for $315 million in 2017. One can speculate that this move was to maintain the market dominance in the secondary hyperparathyroidism in patients undergoing hemodialysis field. Etelcalcetide (Parsabiv) was approved in 2016. The peptide is administered intravenously 3 times a week. Ac-*D*Cys (Cys)-*D*Ala-*D*Arg-*D*Arg-*D*Arg-*D*Ala-*D*Arg-NH_2_.

The peptide reduces parathyroid hormone (PTH) better than Sensipar in hemodialysis patients, with 11.4% more patients being below the threshold clinical level than with the small molecule drug (Patel and Bridgeman, 2018). Etelcalcetide works so well that one of the major side effects is hypocalcaemia (Block et al., 2016). Unfortunately, the observed prolonged QT interval upon peptide treatment cannot be disentangled from the low serum calcium concentration. Another problem is that the peptide was studied only in hospitals, so no early data for at-home hemodialysis, for children and peritoneal dialysis (where oral non-peptide works well) are available. It is also unclear whether the PTH reduction eliminates actual clinical symptoms (bone fracture rate, cardiovascular morbidity, Pereira et al., 2018). Health insurance involvement did not help peptide sales either. Parsabiv sales reached $186 million in the second quarter of 2020 but dropped to $71 million in the same period of 2021. With Etelcalcetide’s inclusion in the US end-stage renal disease bundled payment system, dialysis clinics rapidly implemented new treatment protocols, switching from Etelcalcetide to a generic version of oral Cinacalcet. To make everything worse, a new non-peptide competitor started to dominate the market.

Calcifediol (Rayaldee) is prescribed for hyperparathyroidism secondary to renal impairment (Sprague et al., 2017). The molecule is 25-hydroxyvitamin D3, approved in 2016. It is a prohormone of the active ingredient, calcitriol that binds to the vitamin D receptor in target tissues and activates vitamin D responsive pathways resulting in increased intestinal absorption of calcium and phosphorus and reduced parathyroid hormone synthesis. Rayaldee dosing is 30 μg tablets administered orally once daily at bedtime. At this point, Rayaldee is upcoming and is very popular. The overall Drugs.com rating is 7.9, with 57% of patients reporting positive feelings and 0% negative. Annual sales reached $35 million in 2022 and are constantly increasing. Unlike for Cinacalcet, no lower cost generic is available. Apparently, the impact of Etelcalcetide to human health will never reach its originally anticipated potential.

### How did textbook examples fare so far?

The end of the last century was not good for polyamide-based drugs. If drug developers had followed the infamous, and now widely criticized, “Pfizer rule of five” or “Lipinski’s rule of five,” peptide drugs would have never made the market (Muller-Kuhrt, 2003; Love, 2021). Luckily, pioneering and innovative biotechnology companies swam against the current and developed peptide therapeutics that served as encouragement for further oligoamide drug development. The following three examples were highly inspirational for peptide scientists, at least for the authors of this review article. Let’s take a look how they managed to keep peptide drugs viable alternatives to small molecules over time.

One of the first designer peptide drugs that received considerable public interest was Fuzeon (Enfuvirtide, T-20) which was approved in 2003. It binds to human immunodeficiency virus (HIV) gp41 and competitively inhibits the energy supplying six-helix viral bundle for cell fusion (Wild et al., 1993). While Fuzeon was an eye-opener and switched the negative public view against peptides as possible therapeutics, it still suffers from the activity and administration limitations of early peptide drugs. It is a peptide that mimicks the HR2 region of gp41 containing only natural amino acid residues and has to be subcutaneously injected twice daily. Its structure is Ac-Tyr-Thr-Ser-Leu-Ile-His-Ser-Leu-Ile-Glu-Glu-Ser-Gln-Asn-Gln-Gln-Glu-Lys-Asn-Glu-Gln-Glu-Leu-Leu-Glu-Leu-Asp-Lys-Trp-Ala-Ser-Leu-Trp-Asn-Trp-Phe-NH_2_. Primary HIV-1 virus isolates exhibit variable susceptibilities to Enfuvirtide and thus resistance induction is a major concern (Poveda et al., 2005). Fuzeon is considered a second-line anti-retroviral agent (Qian et al., 2009). The authors of this article have always been concerned about optimization of peptide drug leads. For a 36-residue peptide, it is nearly impossible not to find a better analog in terms of covering extended chemical space, stability and pharmacodynamic properties. When we posed the personal question to a Trimeris lead scientist in 2004 whether they identified a more potent analog, the answer was yes, but investors and drug developers did not want to spend additional money for a potentially better version. The official answer is that a second-generation analog, Tifuvirtide, was found to be 10-times more active (Lalazari et al., 2005) but clinical development was discontinued due to formulation issues. A few additional peptidic fusion inhibitors were identified but never approved.

The significantly larger resources of big pharma usually allows the systematic improvements of small molecule peptide drug competitors and can develop a successful formula. Maraviroc (Selzentry, Celzentry in Europe) from Pfizer is a non-peptide inhibiting HIV gp120 attachment to binding to the CCR5 co-receptor. It is administered twice daily as an oral solution. On the way to approval in 2007, a number of problems associated with the original lead (Dorr et al., 2005) had to be solved by one-by-one medicinal chemistry optimization (Armour et al., 2006): a) high lipophilicity; b) side reaction as a potent CYP 2D6 inhibitor; c) a tropane-based analog turned to be a hERG ion channel inhibitor; and d) two additional structural changes were introduced leading to the final drug composition. Maraviroc works only for 50%–80% of patients, but around 2010 had generated 6-7 times more money than Enfuvirtide (these are the only two approved HIV fusion inhibitors). The monthly patient costs of the peptide are more than double that of the non-peptide.

Although we focus here on peptide drugs acting on well-defined targets, we cannot leave this review without briefly mentioning Prialt (Ziconitide). Approved in 2004, it was seminal to establishing positive public opinion to peptide drugs. Prialt is derived from the cone snail *Conus magus*, comprises 25 amino acids with three disulfide bonds and it acts as an N-type calcium channel blocker for the management of chronic pain (Miljanich, 2004). Its primary structure is H-Cys-Lys-Gly-Lys-Gly-Ala-Lys-Cys-Ser-Arg-Leu-Met-Tyr-Asp-Cys-Cys-Thr-Gly-Ser-Cys-Arg-Ser-Gly-Lys-Cys-NH_2_ (disulfide bonds Cys1-Cys16, Cys8-Cys20, and Cys15-Cys25). Remarkably, Ziconitide has no approved competitor of any drug class. To date, Ziconotide is the only calcium channel blocking peptide approved for use by the FDA. Prialt reached a textbook example status among pain medications because of the lack of signs of dependence and of no apparent tolerance. Ziconotide is administered intrathecally over 1 hour. Given the intrathecal administration and low membrane permeability due to its size, Ziconotide is expected to remain primarily in the cerebrospinal fluid (CSF); plasma levels, where detected, remain constant up to 9 months following administration (McGivern, 2007). For cancer pain, morphine is also recommended as first line therapy, but the consensus is to use Prialt without hesitation unless contraindicated (Deer et al., 2019). However, for its long standing as cancer pain killer, a remarkable 73 tons of morphine for cancer was produced in 2023 (14% of 523 tons total, van Zee, 2009) compared to Prialt which sold for a total of $27 million in 2017. To be fair to morphine, monotherapy with the peptide costs 2.5 times of that of the opioid (Lambe et al., 2023).

Our last example has a personal connection to these authors. We have provided a few examples for replacing approved peptide drugs with small molecule therapeutics. Due to our interest in adipokine receptor modulators namely, leptin and adiponectin receptor agonists and antagonists (Otvos et al., 2009; Otvos, 2019), we submitted tens of public and private grant proposals for replacing Tesamorelin against lipodystrophy with a leptin receptor agonist peptide drug but to no avail. Apparently, the benefits of Tesamorelin would not justify the development of any peptide competitor. Tesamorelin (Egrifta) is a human growth hormone-releasing factor (GRF) analog for lipodystrophy induced by antiviral therapy in AIDS patients (Lake et al., 2021) and has the primary structure Hexenoil-Tyr-Ala-Asp-Ala-Ile-Phe-Thr-Asn-Ser-Tyr-Arg-Lys-Val-Leu-Gly-Gln-Leu-Ser-Ala-Arg-Lys-Leu-Leu-Gln-Asp-Ile-Met-Ser-Arg-Gln-Gln-Gly-Glu-Ser-Asn-Gln-Glu-Arg-Gly-Ala-Arg-Ala-Arg-Leu-NH_2_. Approved in 2010, it is still produced by the same biotechnology company which developed it (Theratechnologies). Tesamorelin is administered once daily by subcutaneous injection. The peptide has no competitor for HIV-related lipodystrophy. For generalized lipodystrophy metreleptin (Myalept), a recombinant leptin derivative can be used but even this competitor is a biologic medication.

Engrifta fills a hole in lipodystrophy drugs. The 16:0 FDA approval, a sign of desperate need for lipodystrophy treatments, came despite pronounced anabolic effects and even more evident adverse effects (liver and kidney findings, anaemia, clinical chemistry changes, organ weight effects) observed in dogs after repeat daily subcutaneous injections, which were all attributed to prolonged exposure to supraphysiological levels of the growth hormone and/or the insulin-like growth factor (IGF)-1 (Ferdinandi et al., 2007). Despite these side effects, this medication is truly needed, documented by the sales volume in 2021 of $41 million and $50 million in 2022 indicating constant, if not increased, usage even a dozen years after approval. While Tesamorelin is not a blockbuster, it is an especially useful addition to the human pharmaceutical arsenal helping 1,000–2,000 patients annually and producing a constant revenue stream for the developers. Most of us peptide therapeutics designers and developers would be very happy to produce a drug like that.

### Some additional approved peptide drug targets–and the lack thereof

While discussing the merits of peptide therapeutics, experts pointed out a few additional successful peptide drugs on the market and several others in late clinical development stage. Since more than half of drug candidates passing phase II trials fail in phase III (van Norman, 2019), listing these peptides would be premature in the context of this review article. However, some additional approved peptide drugs are worth briefly mentioning. Pasireotide is a hexamer homodetic cyclic somatostatin analog approved in 2012 for the treatment of Cushing disease caused by a tumor or excess growth (hyperplasia) of the pituitary gland (Pivonello et al., 2019). Pasireotide was included in the reference review article (Wang et al., 2022) as was another anti-tumor peptide, Degarelix, approved in 2008, an antagonist of gonadotropin-releasing hormones (GnRH) for the treatment of advanced prostate cancer. It distinguishes itself among the sea of GnRH agonists used for androgen deprivation therapy (Sciarra et al., 2016). What was approved before 2000 (exactly in 1987 and accordingly not listed) is another prostate (and breast) cancer treatment, Goserelin, a synthetic analog of the luteinizing hormone-releasing hormone that acts by reducing secretion of gonadotropins from the pituitary (Bolla et al., 1997). Goreselin earned the fame of being a member of the World Health Organization’s List of Essential Medicines. For targeting diseases mostly affecting women, two remarkable peptide drugs are Abaloparatide, utilized in osteoporosis management and Elagolix, employed in the relief of endometriosis-associated pain and the management of heavy menstrual bleeding. While Abaloparatide is an N-terminal analog of parathyroid hormone-related protein (PTHrP) 4 and an agonist at the parathyroid hormone type 1 (PTH1) receptor (Pioszak et al., 2009, first approval date: 2017), Elagolix, approved in 2018, is yet another gonadotropin releasing hormone receptor antagonist and is used to treat moderate to severe pain in endometriosis. It is a rare orally-administered peptide drug that inhibits endogenous GnRH signaling by binding competitively to GnRH receptors in the pituitary gland (Leyland et al., 2021).

Remarkably, what is missing from this list are antimicrobial peptides (AMP). Although as many as 3,000 AMPs have been reported and characterized, only 7 are approved by the FDA (gramicidin D, daptomycin, vancomycin, oritavancin, dalbavancin, colistin and telavancin), all acting on bacterial membranes, and none of them are classical peptide therapeutics regarding their structure and all lack of specific molecular targets (Chen and Lu, 2020). Although early preclinical studies show quite promising results against resistant bacterial infections (Ostorhazi et al., 2018), in late preclinical assays and clinical trials AMPs failed to show sufficient antimicrobial activity (Silver, 2011). Their poor performance may derive from differences between the clinical setting and the screening conditions (Luo et al., 2023). Declining current public investment in new antimicrobial drugs does not help the situation either (World Health Organization, 2020).

An interesting aspect of “de-orphanization” of rare G-protein-coupled receptors (GPCR) with peptide ligands is to expand peptide drug coverage (Hauser et al., 2020). In fact, we took exactly the opposite approach. While our adiponectin receptor (hardly an orphan GPCR) targeting peptide agonist, ADP 355 (Otvos et al., 2011), did not show pronounced activity in an initial phase 3 clinical trial against the multibillion dollar dry eye disease, it shows remarkable efficacy in animal models of orphan diseases such as Duchenne muscular dystrophy syndrome (Dubuisson et al., 2023). Phase 1 clinical trials of a systemic formulation (as opposed to the eyedrop) will start in Q1 2024.

## Concluding remarks

It is undeniable that peptides, natural biomolecules, have contributed enormously to the advance of chemical and biological science and have profoundly impacted the development of the modern pharmaceutical industry. Because of their high specificity and low toxicity profiles in humans, designer peptide drugs are now sought-after alternatives to small molecule therapeutics and a major focus for creating next-generation products (Otvos and Wade, 2014; Fosgerau and Hoffmann, 2015). Despite the challenges with their development as outlined above and previously (Otvos and Wade, 2014), today, the peptide market is growing nearly *twice* as fast as the overall pharmaceutical market due to an increased number of therapeutic targets and improved delivery methodologies. There are now 80 therapeutic peptides on the market, 200 in clinical phases, and 600 in advanced pre-clinical stages (Henninot et al., 2018). Worldwide sales of peptide drugs are projected to reach a staggering $75 billion by 2028 (Arora, 2022). It is clear that their promise is being increasingly realized in the pharmacy and that with further inevitable advances in drug design, formulation and delivery systems, they will continue to be critically important alternatives to small molecules for therapy. The current trend of designing a road map to help identify the best course for selecting drug targets in light of recent clinical developments would be a useful set of guidelines for even more successful competition with traditional drug classes.
